# Lossless Compression of Human Movement IMU Signals

**DOI:** 10.3390/s20205926

**Published:** 2020-10-20

**Authors:** David Chiasson, Junkai Xu, Peter Shull

**Affiliations:** State Key Laboratory of Mechanical System and Vibration, School of Mechanical Engineering, Shanghai Jiao Tong University, Shanghai 200240, China; dchiasso@sjtu.edu.cn (D.C.); abcyxjk@sjtu.edu.cn (J.X.)

**Keywords:** codec, compression, human movement, IMU, kinematic data

## Abstract

Real-time human movement inertial measurement unit (IMU) signals are central to many emerging medical and technological applications, yet few techniques have been proposed to process and represent this information modality in an efficient manner. In this paper, we explore methods for the lossless compression of human movement IMU data and compute compression ratios as compared with traditional representation formats on a public corpus of human movement IMU signals for walking, running, sitting, standing, and biking human movement activities. Delta coding was the highest performing compression method which compressed walking, running, and biking data by a factor of 10 and compressed sitting and standing data by a factor of 18 relative to the original CSV formats. Furthermore, delta encoding was shown to approach the a posteriori optimal linear compression level. All methods were implemented and released as open source C code using fixed point computation which can be integrated into a variety of computational platforms. These results could serve to inform and enable human movement data compression in a variety of emerging medical and technological applications.

## 1. Introduction

Many emerging technological and medical applications rely on real-time human movement inertial measurement unit (IMU) signals as crucial components, including virtual reality [1], autonomous navigation [2], Internet of Things [3], activity monitoring [4,5], physical therapy [6,7], and human performance science [8,9] among others. These applications have been enabled by the recent explosion of inexpensive inertial-based sensors (IMUs) along with the increase in mobile computational power to process these data in real time. The human movement IMU signal represents a nascent field of multimedia processing which is starkly under-developed compared to the existing maturity of text, audio, and visual-type signal processing methods. This is evidenced by the lack of standards and tools for handling movement data. To enable these emerging applications, efficient and standard methods for representing and processing movement signals are needed. Compression can be a crucial component of this missing toolset, as it improves situations of limited transmission bandwidth and limited storage space.

Some human movement measurement applications are likely to encounter technical limitations related to bandwidth and storage space. A back of the envelope calculation for measuring full body kinematics of a single subject at 500 Hz [10] × 15 segments × 9 axes × 32 bits = 2.16 Mbps. This is approximately the same bandwidth as streaming high definition video on modern consumer platforms [11]. It also exceeds the throughput of common wireless sensor network technologies, such as Bluetooth and Zigbee. A physical therapist wanting to monitor the kinematics of their patients around the clock could find themselves producing terabytes of information every day, well beyond what is economically feasible to store.

**Figure 1:**
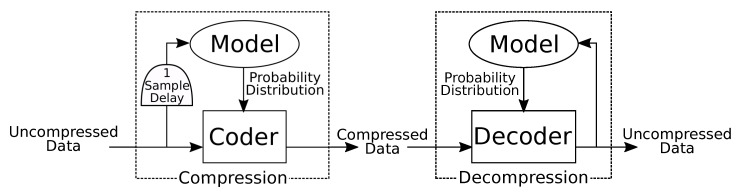
Generalized compression and decompression. Modeling and coding are independent. The model is mathematically identical for compression and decompression. The coder and decoder perform inverse operations parameterized by the probability distribution.

Compression seeks to represent information in a space efficient manner. This is generally done by exploiting spatio-temporal redundancy, correlation, and smoothness [12]. A lossless compression algorithm can be divided into two components: modeling and coding/decoding (Figure 1) [13]. The model incorporates prior understanding of the signal class to be compressed. It estimates a probability mass function which represents the likelihood of occurrence for each possible input symbol. A dynamic model is one which changes its probability estimates after new input symbols are received. The model is sometimes described as a transformation, which refers to some reversible operation which changes the signal into a lower entropy or easier to predict form. The coder uses the probability mass function produced by the model to compute a unique variable length code for each possible symbol. Short codes are assigned to likely symbols, and long codes are assigned to unlikely symbols such that the average length of the compressed signal is minimized. The decompressor uses an identical model to provide the same probability mass function to the decoder, which is used to revert each code back into the original symbol. If the model is dynamic, this output is used to update the model for future predictions.

The coding component is well understood. The theoretical limit of coding performance on a signal with a known probability distribution is given by Shannon’s noiseless coding theorem [14] as first-order entropy:

$$H=-\sum_{i}P_{i}log_{2}P_{i}$$

While Shannon developed several efficient coding techniques, the first optimal technique was developed in 1952 by Huffman [15]. Huffman’s technique was proven optimal, but it made the assumption that output code must be an integer number of bits, which prohibited it from reaching Shannon’s limit under certain conditions. Arithmetic coding was developed to address this deficiency, showing better performance, particularly in high compression situations [16]. Another technique, Golomb coding and the Rice special case, has been widely adopted in industry because of the efficient binary implementation, and the ability to encode online without needing a preliminary pass through the data to compute the probability distribution [17,18]. Due to the many efficient and optimal techniques available, some consider coding to be a solved problem [19].

Modeling, on the other hand, must be revisited for each signal class and application. Due to the pigeon hole principal, no algorithm can compress every possible input [20]. Each model must make an implicit decision about the class and scope of signals that will be compressed. A model which accurately predicts signals from one class may not predict signals from a different class. To the authors’ knowledge, no previous work has directly addressed the modeling problem for human movement IMU signals.

This study thus explores predictive models applied to the human movement signal as quantified by 6-axis IMUs. Proposed models are restricted to those that are real-time, node independent, and lossless. A corpus of representative human movement IMU signals was selected to demonstrate the compression performance of each model. To put the performance in context, several traditional representation formats were selected. The compression performances of these traditional formats provide a floor for the performance of a useful compression method. Finally, the optimal linear predictive models were computed numerically while utilizing full knowledge of the signals in the corpus. The compression performances of these optimal models provide an upper boundary for the performance of our proposed methods. We hypothesized that compression ratios would be comparable to those achieved in lossless audio compression. We also hypothesized that the best performing codec would utilize some cross axis correlation and be informed by physics based models.

## 2. Methods

This section details the seven proposed compression methods, and then explains the three traditional methods and two optimal methods which are used to provide context as lower and upper bounds respectively. Next, the corpus of signals used for performance assessment is introduced, followed by implementation details and data analysis methodology.

With the exception of the traditional data representation formats, all compression methods in this work are presented as predictive autoregressive linear models. The predictive model estimates the current sample given past input. The difference between the model prediction and observed sample is referred to as the residual signal. All compression methods encode this residual signal using Golomb–Rice coding to produce the final compressed data. Golomb–Rice coding is computationally efficient on binary base computational platforms, and has been shown to approach optimal coding if the input signal is geometrically distributed [17,18].

### 2.1. Proposed Compression Methods

Several restrictions are placed on the methods considered in this study. First, a viable algorithm must be causal. This is a basic requirement for an algorithm to be implemented in a real-time application. We also chose to only consider algorithms with zero filter delay. Since our sensors operate at a relatively low sampling frequency of 60 Hz, a delay of one sample would be 16 ms, which is significant for modern information networks.

All algorithms considered in this paper are node independent. The model for each signal considers at most the information from the six co-located signals, including its own past input. While it is possible that utilizing inter-node correlation could produce better compression ratios, especially if a human biomechanics model were introduced, such an approach would limit the usefulness of said algorithm to a specific placement of nodes on the body.

Only lossless compression methods are considered in this study. The reason for this is that designing a lossy compression method requires a well-defined distortion criterion [12]. This criterion is a value judgment about what type and magnitude of distortion is acceptable for the compressed signal. Defining a distortion criterion for audio and visual signals, while not trivial, is certainly tractable, as there is generally a single, well-defined application of the signals, namely, consumption by the human ear and eye [21,22]. Other signal classes such as text and binary data have sensitive applications that cannot tolerate any error in the signal, and thus lossy compression methods are not considered. The human movement IMU signal on the other hand has an array of applications, each of which may have different requirements for acceptable and unacceptable distortions of the movement signal. This is problematic, as a single distortion criterion will likely not be ideal for all applications. Without choosing a criteria, discarding of any portion of information is of course arbitrary.

In practice, the implementation of a strictly lossless algorithm turns out to be non-trivial. This is because the standard for floating point computation IEEE 754 [23] is not sufficiently stringent to guarantee identical results on various implementations [24,25]. For example, rounding is permitted to differ slightly between two computation platforms, or the same computation platform at two points in time. Additionally, a compiler or interpreter which processes the source code for an algorithm implementation will often utilize mathematical properties such as commutativity to optimize computation. This may result in different machines performing floating point operations in a different order, which could lead to differing results even if each rounding operation was well defined by IEEE 754. To avoid both of these scenarios and guarantee identical results across diverse computational platforms, all algorithms in this study were implemented using integer operations and fixed-point 16.16 precision.

The following lossless compression methods are proposed in this study:

- **Delta encoding** encodes the difference between each successive sample. The current sample is predicted to be equivalent to the previous sample $\hat{x}\left[n\right]=x\left[n-1\right]$ so that the residual e[n]=x[n]−x[n−1] is encoded. Despite the name, delta encoding is discussed in this context as a modeling technique which predicts that a signal is constant. If a signal varies slowly with time, the residual will be close to zero and readily compressed with coding techniques such as Golomb–Rice coding. This method can be considered 0-order polynomial regression.
- **Linear extrapolation**: The current sample is estimated as a linear extrapolation from previous samples—also known as first-order polynomial regression. Linear extrapolation from a regression of the past two samples results in the estimator: $\hat{x}\left[n\right]=2x\left[n-1\right]-1x\left[n-2\right]$.
- **2nd to 5th order polynomial regression**: These methods assume that the signal is a polynomial which is estimated from a least squares regression of past samples. If the sampling period is fixed, the polynomial coefficients b may be found via the problem:
  $$\begin{array}{cccc} \; & \underset{b}{\mathrm{minimize}}\; & \; & \sum_{i=1}^{p}{\left(x\left[n-i\right]-\sum_{j=0}^{d}b_{j}{\left(i-p-1\right)}^{d-j}\right)}^{2} \end{array}$$
  where *d* is the polynomial order and p>d is the number of past samples included in the regression or the order of the resulting predictive model. This polynomial is then extended to get a prediction of the current sample.
  $$\begin{array}{c} \hat{x}\left[n\right]=\sum_{i=0}^{d}b_{d-i}n^{i} \end{array}$$
  Without loss of generality, we can take n=0 and rewrite our optimization problem as
  $$\begin{array}{cccc} \; & \underset{b}{\mathrm{minimize}}\; & \; & {∥x-Cb∥}_{2}^{2} \end{array}$$
  where $C\in R^{p\times d+1}$ is composed of $c_{ij}={\left(i-p-1\right)}^{d-j}$. This has the well known solution $b={\left(C^{T}C\right)}^{-1}C^{T}x$. Our prediction for the current sample can now be written as
  $$\begin{array}{c} \hat{x}\left[0\right]=b_{d}=e_{d}{\left(C^{T}C\right)}^{-1}C^{T}x \end{array}$$
  where $e_{d}$ is the standard basis vector. Since C is independent of our signal, the vector of filter coefficients $e_{d}{\left(C^{T}C\right)}^{-1}C^{T}$ can be precomputed for a given polynomial order *d* and filter length *p*. Surprisingly, this allows us to compute high order polynomial extrapolations as a simple linear combination of past samples or an autoregressive model of order *p*.
- **Spline extrapolation**: A spline is the minimum curvature piecewise polynomial which connects a set of points. It is commonly used for interpolation, namely, computer graphics smoothing. This method was selected as splines are known to avoid Runge’s phenomenon, which is witnessed when extrapolating higher order polynomials. Results from the cubic spline with natural boundary conditions are presented in this paper. The cubic spline is a piecewise cubic function:
  $$f_{i}\left(n\right)=a_{i}n^{3}+b_{i}n^{2}+c_{i}n+d_{i}\;n\in \left(n_{i},n_{i+1}\right)$$
  which is restricted as smooth
  $$\frac{d}{dn}f_{i}\left(n_{i+1}\right)=\frac{d}{dn}f_{i+1}\left(n_{i+1}\right)\;$$
  and passes through the set of *p* past samples.
  f(n)=x[n]
  By choosing natural boundary conditions,
  $$\frac{d^{2}}{dn^{2}}f_{1}\left(n_{1}\right)=\frac{d^{2}}{dn^{2}}f_{p-1}\left(n_{p}\right)=0\;$$
  we can extrapolate the spline to predict the next sample.
  $$\hat{x}\left[n_{p+1}\right]=f_{p-1}\left[n_{p+1}\right]$$
  Like polynomial regression, spline extrapolation is also an autoregressive linear model.

### 2.2. Traditional Representation Formats

To provide a lower bound for the useful performance of compression methods, several traditional data representation formats were chosen for reference. In this study, the baseline data format (exhibiting a compression ratio of one) is comma separated values (CSV), a simple text based format which is the de facto standard for storing sensor information. The performance of each compression method was assessed by computing the compression ratio (CR) relative to the CSV via the following formula:

$$CR=\frac{\mathrm{size}\mathrm{of}\mathrm{CSV}\mathrm{file}}{\mathrm{size}\mathrm{of}\mathrm{compressed}\mathrm{file}}$$

In total, the following three traditional data representation formats were chosen to provide context for our results:

- **CSVL** Text-based format considered the de facto standard. CSV files are ANSI encoded and formatted to have a constant length sample format to eliminate a source of randomness in our CR computation. Due to this decision, binary format will have the same CR regardless of data properties.
- **Binary**: The optimal fixed size format. In our corpus, every sample is two bytes. This would be the optimal compression if each sample were an IID random variable uniformly distributed across the sample space.
- **ZIP compression of CSV**: ZIP is a general purpose file compression format integrated into all major computer systems. ZIP was executed using the DEFLATE method [26] and a compression level of 6.

### 2.3. Optimal Linear Compression

To complete the context for our proposed compression methods and provide an upper limit on compression performance, we numerically computed the optimal linear predictive model for our data. To do so, we formulated our model as an autoregressive process of order *p* and define the prediction error or residual signal as:

(1) $$e\left[n\right]=x\left[n\right]-\sum_{k=1}^{p}a_{k}x\left[n-k\right]$$

where $a_{k}$ is the linear contribution of sample *k* in the past to our current prediction. Equation (1) can expressed be in concise matrix notation as:

e=x−Xa

For our application, we are interested in the residual signal e which can be encoded into the minimum number of bits. To compute this, we considered the effect of Golomb–Rice coding on our residual signal. A Golomb–Rice encoding first splits each residual e[n] into a quotient and remainder portion:

$$q\left[n\right]=\left\lfloor \frac{e[n]}{2^{m}}\right\rfloor \;\mathrm{and}\;r\left[n\right]=e\left[n\right]-q\left[n\right]*2^{m}$$

⌊·⌋ denotes the floor operation and $m\in N_{0}$ is the Golomb–Rice order which is selected based on signal statistics [27,28]. The remainder r[n] is truncated binary encoded at a fixed size of *m* bytes, while the quotient q[n] is unary encoded, requiring q[n]+1 bits. The size in bits of each Golomb–Rice encoded element of the residual signal is thus:

$$m+\lfloor \frac{e[n]}{2^{m}}\rfloor +1$$

If we relax our rounding operation, the size of each element can be approximated as a affine function of e[n]. The total compressed size for a signal of length *l* has an approximate size:

$$l+lm+2^{-m}\sum_{n=0}^{l}e\left[n\right]$$

Minimizing this value with *l*-1 normalization is equivalent to the optimization problem:

(2) $$\begin{array}{cccc} \; & \underset{a}{\mathrm{minimize}}\; & \; & {∥x-\mathrm{Xa}∥}_{1}+\lambda {∥a∥}_{1} \end{array}$$

The key takeaway is that compression is proportional to the total absolute prediction error of the model, and not the squared error. *l*-1 normalization is used since it encourages sparsity in the model. Sparsity is desirable in this application, as it reduces the quantization error of the model coefficients and reduces the fixed-point arithmetic error during execution. Since this problem is convex, it can readily be solved with a variety of numerical solvers. For this study, Python bindings for the splitting conic solver were used [29,30,31].

If problem (2) is solved considering past history of each stream, then the model for each axis of accelerometer and gyroscope can be computed independently. This model will be referred to as the optimal autoregressive model (AR). However, if we also take into account the past history of other axes and sensors, then the model can account for any cross-correlation which may result from the interrelated nature of rotation and orientation information. The residual signal (1) can be rewritten using this expanded model for stream *i* as:

$$e_{i}\left[n\right]=x_{i}\left[n\right]-\sum_{j=1}^{s}\sum_{k=1}^{p}a_{i,j,k}x_{j}\left[n-k\right]$$

where $a_{i,j,k}$ is now the linear contribution of the sample *k* in the past of stream *j* to our current prediction of stream *i*. Solving problem (2) with this expanded system will be referred to as the optimal multivariate autoregressive model (MVAR). Note that both AR and optimal MVAR models are non-causal and expensive to compute, and are thus disqualified as a proposed method. Instead, they serve as upper-limit reference points for the evaluation of proposed methods.

### 2.4. Movement Data

**Figure 2:**
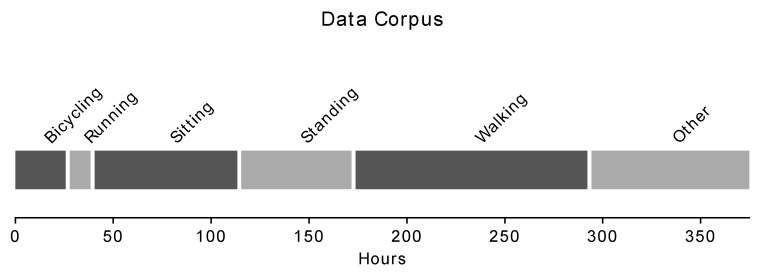
Composition of data in the Human Gait Database. All dimensions of all sensors are counted, i.e., one minute of subject testing generates 36 min of sensor data. “Other” activities consist of walking up and downs stairs, standing up, sitting down, standing on an elevator, and sitting in a car.

The Human Gait Database (HuGaDB) [32] was selected as a corpus to meaningfully and repeatably demonstrate the performances of various compression methods. HuGaDb is a public dataset of six-axis IMU signals collected from six different body segments (right and left foot, right and left shank, right and left thigh) of 18 healthy subjects performing various movement activities (including walking, running, sitting, standing, and biking) sampled at 60 Hz (Figure 2). This database was selected because it allows the comparison of compression methods for a variety of movement activities. Signals were sampled as 16-bit signed integers. The authors of HuGaDB indicate in their online repository that some of their collected data contain corrupted gyroscope signals. Such data have been excluded from this study. The remaining data were separated by subject, movement activity, trial, and body segment, to produce a total of 1626 separate test cases to compare the performances of proposed compression methods. Each test case contained six time series signals.

### 2.5. Implementation

All compression methods in this study were implemented in the C programming language using fixed-point 16.16 computation. The source code has been released at https://github.com/dchiasson/kinetic_codec. The choice of programming language as well as the restriction to use integer arithmetic allow this code to be incorporated into programs on a diverse array of computational platforms, even those without any floating-point unit.

### 2.6. Data Analysis

**Figure 3:**
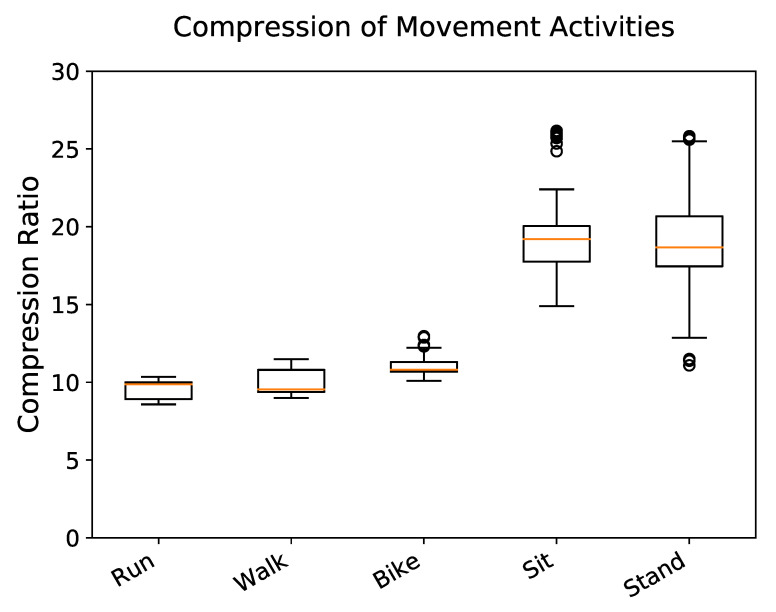
Distribution of compression ratio (original size/compressed size) of each movement activity using delta encoding. Stationary movement activities experience greater compression than active movement activities.

Performance was quantified and compared via standardized methods for comparing classifiers with multiple datasets [33]. To compare the CRs of our proposed compression methods, we used the Friedman Test [34] which is a non-parametric test for significant differences between algorithm performance and considers the ranks of each algorithm’s performance per test case. A non-parametric test is necessary since our corpus does not meet the normal distribution assumption of many parametric tests. This is demonstrated in Figure 3 by the significant separation of active and stationary movement activities. If the null-hypothesis was rejected, we proceeded with the Nemenyi post-hoc test [35] to determine which pairs of methods differ significantly. Instead of directly using compression ratios, the Friedman and Nemenyi tests consider the ranks of compression performance, with one being the best performing and seven being the worst performing for each test case. Statistical analysis of performance is presented for proposed method results over the entire corpus.

Additionally, to determine if a proposed method had significantly different performances on different classes of data the parametric ANOVA test was used [36]. A parametric test may be used in this case since compression performance on each individual movement activity showed a near normal distribution. If the null-hypothesis was rejected, the Tukey HSD post-hoc test [37] was used to determine which pairs of data classes experienced significantly different compression. This statistical analysis is presented for the highest performing proposed method. The statistical level of significance was set to p=0.05.

## 3. Results

**Figure 4:**
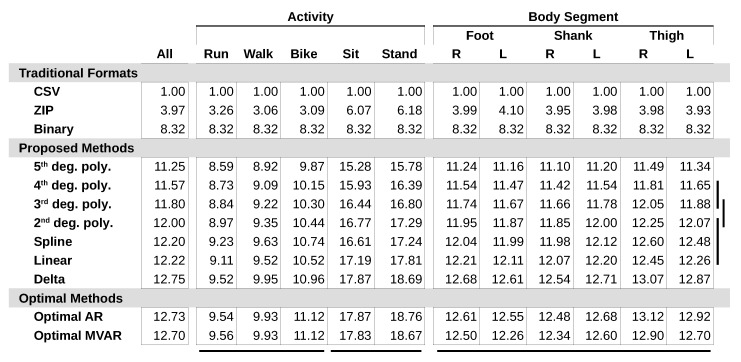
Compression ratios for all methods across movement activity and body segment. Groups of data classes and methods without significantly different compression (p≥0.05) are indicated by bars below and to the right of the table respectively. Specifically, horizontal bars represent statistical analysis of the delta compression method applied to each data class, while vertical bars represent statistical analysis of each proposed method applied to all data.

All proposed compression methods outperformed all traditional methods in size efficiency for every data class and nearly every test case (Figure 4). Delta encoding achieved the highest compression of the proposed methods (CR =12.75), and each higher degree polynomial performed progressively worse; 5th degree polynomial was the worst performing (CR =11.25). The spline method was not found to be significantly different from linear extrapolation or 2nd degree polynomial regression (p>0.52). Significant differences were also not found between 3rd and 4th degree polynomials (p=0.25) or between 2nd and 3rd degree polynomials (p=0.19). The CR of delta encoding approached optimal AR and MVAR model compression for all data classes.

**Figure 5:**
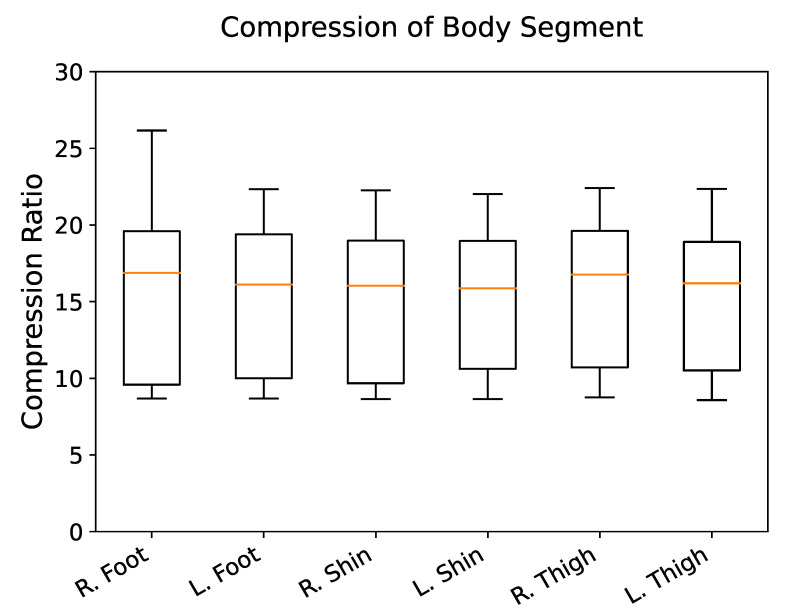
Distribution of compression ratio (original size/compressed size) of each body segment using delta encoding. All body segments achieved similar compression.

Stationary movement activities, such as sitting (CR =17.87) and standing (CR =18.69), were compressed more than active movement activities, such as running (CR =9.52), walking (CR =9.95), and biking (CR =10.96). Significant differences were not found within the stationary movement activity group (p=0.90) or the active movement activity group (p>0.13) (Figure 3). Body segments did not show significant variation in CR (p>0.05) (Figure 5).

## 4. Discussion

**Figure 6:**
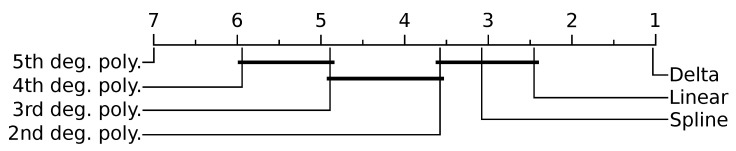
Mean performance ranking of proposed methods compared using the Nemenyi test. Groups of methods that are not significantly different (at p=0.05) are connected. Delta encoding being close to one is due to outperforming all other techniques in nearly every test case. Spline on the other hand was ranked third on average, but ranked higher or lower for some test cases.

Results show that the proposed compression methods result in a significant compression as compared with the traditional representation formats of CSV, ZIP, and binary. Delta encoding was the best performing of the proposed compression methods. It achieved the highest average compression (CR =12.75) and achieved superior compression for nearly all test cases (Figure 6). This aligns with other research often recommending delta encoding as a lossless compression model in other domains [38,39]. Each increasing order of polynomial regression produced slightly worse compression (CR =12.22,12.00,11.80,11.57,11.25). This is likely because higher degree polynomial predictors suffer from poor white noise attenuation [40] causing an effect known as Runge’s phenomenon.

In several test cases, delta encoding slightly exceeded the compression of the optimal reference models. In theory, the optimal linear models should perform as well as or better than delta encoding, since the delta encoding model exists within the domain of optimization problem (2). The occasional inferior performances of the optimal linear models might have been due to quantization error of the model coefficients and increased fixed point error from computational complexity. This explanation is supported by the observation that the MVAR model, which has many more coefficients, often underperforms relative to the AR model, while the opposite would be true in the absence of numeric error. Investigation of the optimal models’ coefficients showed that they were similar to those of delta encoding.

The compression level achieved on each movement activity varied significantly and separated into two distinct groups. The first group–consisting of running, walking, and biking (active movement, CR ≈10) achieved much less compression than the second group–sitting and standing (stationary movement, CR ≈18) (Figure 3). This aligns with our expectations, as higher intensity of movement will likely have more information content.

The MVAR optimal model and the AR optimal model achieved similar performances. This fails to suggest a linear relationship between the various axes of accelerometer and gyroscope information and fails to support our hypothesis. While we would expect a relationship between rotation, as measured by the gyroscope, and orientation of the gravity vector, as measured by the accelerometer, this is not a linear relationship and thus was not captured by the proposed models. This study does not preclude the possibility of a non-linear model to successfully exploit such a relationship.

### Limitations and Future Work

This study is limited by the sensing hardware used for data collection, the placement of sensors on lower body segments, and the exploration of only linear models. The compression ratios presented in this paper are intended to demonstrate the relative difference between compression methods and may not be representative of the absolute CR experienced in other applications. There are many other factors which can affect the compression ratio which were not explored in this paper. Namely, sensor differences of precision, noise, bias, and sampling rate are expected to have a large impact on the CR achieved. That being said, our corpus consisted of low-cost consumer grade IMUs at a low sampling rate, and the authors would expect many applications to experience significantly higher compression than presented here if higher sampling rates or higher quality sensors are used.

The corpus chosen for demonstrating performance in this work allowed us to explore the effect of movement activity and body segment on compression. However, it could be improved for this purpose by including diverse hardware and higher sampling rates which would be more representative of applications. Signals from more body segments and magnetometer signals should also be included in an ideal corpus.

In future work, the proposed methods described in this paper could be improved to dynamically detect the optimal Golomb coding order and to recover from dropped packets. Dynamic linear models have shown promising results in similar applications [38], and non-linear models can also be explored. The community would also benefit from a standardized format for representing IMU data, as well as distortion criteria for the various applications of the human movement IMU signal. This would pave the way for the development of lossy compression methods.

## 5. Conclusions

This work explored methods for the compression of human movement IMU signals. For the corpus selected, delta encoding was found to achieve near-optimal linear compression, and outperformed traditional methods for all movement activities and body segments. This suggests that delta encoding can be used to reliably compress the IMU human movement signal in many situations. This result could be used to significantly decrease the required transmission bandwidth and storage space required for the implementation of medical and technological human movement applications without any loss of quality.
